# Mushroom Poisoning

**Published:** 1907-07-13

**Authors:** 


### SPECIAL ARTICLE.

### MUSHROOM POISONING.

Mushroom poisoning is not absolutely common in this country, but cases occur every year, especially in rural districts, and occasionally the trouble is epidemic. The same applies to France and other European countries, and to America. The poisonous fungi and their effects are very similar in different places, and they have recently been described afresh from a clinical point of view by William W. Ford, M.D., D.P.H., Associate Professor of Bacteriology and Lecturer on Hygiene in Johns Hopkins University. The commonest fungi to cause toxic symptoms are *Amanita phalloides*, *Amanita muscaria*, *Amanita pantherina*, *Morchella esculenta* or Morells, and there are a few that are less common. It is obvious that the fungi that will most frequently be eaten are those which are least unlike ordinary mushrooms; poisoning by the bright scarlet poisonous fungi, for example, is very rare.

### Poisoning by Amanita Phalloides.

*Amanita phalloides*, known commonly as "white" or "deadly Amanita," with the closely allied species *Amanita verna*, is the cause of by far the majority of fatalities from mushroom eating. It is known by a dozen various Latin names; in France it is called "l'oronge ciguë," "l'oronge blanche ou citronnée," "l'oronge ciguë jaunâtre," and "l'oronge souris." The Germans call it popularly "Knolblätterschwamm." The species indicated by these various names has a characteristic appearance, and is readily recognised by ordinary collectors of fungi. It usually grows to a height of five to seven inches, and it consists of a base or expanded cup (the poison cup), upon, or more properly speaking, within which rests the stalk. The latter is surrounded by an expanded top or pileus to whose under surface are attached the gills covered with white spores. The plant is pure white in colour, with the exception of the pileus, the upper surface of which varies from a china-white to an amber or pale yellow. Occasionally the top is greenish. The cup, the stalk, the under surface of the pileus, and the gills are always pure white, with the possible exception of a few flakes of yellow rarely seen on the stem. The pileus is usually smooth, but at times delicate flakes or scales easily removed by brushing lightly with the fingers, are found on the upper surface. The *Amanita verna*, regarded by many mycologists as merely the spring form of *Amanita phalloides*, differs from the latter only in size and colour. This form is always pure white, has few or no scales on the pileus, and grows to a height of but four to five inches. Much smaller forms are very abundant in the early spring, possibly the most common variety being a plant only about 2½ to 3 inches tall, with a pileus measuring 1 to 1½ inches in diameter. In all these species the colour of the spores may easily be ascertained by allowing the pileus to rest gills downward upon a sheet of paper. After the lapse of three or four hours the spores are deposited upon the paper in a design representing the arrangement of the gills, and with a colour characteristic for each species. Neither *Amanita verna* nor *Amanita phalloides* is difficult to recognise, and both forms are abundant.

### The Symptoms.

The clinical symptoms of this form of intoxication are characteristic. After an incubation period of six to fifteen hours there is a sudden attack of extreme abdominal pain, accompanied by vomiting and diarrhœa. Vomitus and stools consist of undigested food with much blood and mucus. Anuria is usually present, and rarely constipation is seen. Hæmoglobinuria has never been described, and in many cases specific statements are made as to the lack of colouring matter in the urine. Paroxysms of pain and vomiting alternate with periods of remission, the extreme suffering producing a Hippocratic facies, characterised by the French as "la face vultueuse." The loss of strength is rapid and excessive. Jaundice, cyanosis, coldness of the skin, especially of the extremities, develop within two or three days, followed by profound coma and death. Ocular symptoms and convulsions are rarely noted, but both may occur, the convulsions appearing as a terminal event more commonly in children than in adults.

### The Course and Prognosis.

The disease lasts four to ten days. If large quantities of the fungus have been eaten a very profound intoxication develops, and death may occur within forty-eight hours. The mortality in this form of intoxication is very high, varying from 60 to 100 per cent. in different epidemics, and death may follow the consumption of surprisingly small quantities. Plowright, for instance, has reported the death of a child of ten years from eating one-third of the top of a small plant, and there are numerous deaths reported from eating one or two good-sized specimens.

Cooking these fungi does not destroy their toxicity, the majority of accidents occurring with well-cooked material. The strength of the poisonous principles is apparently lessened, however, since smaller quantities of the raw plant can produce a fatal result than when the plants have been thoroughly heated.

The cases reported by Pfromm are so typical of nearly all the epidemics that they may well be quoted as representing the usual course of events. A family of Italians collected one Sunday a large quantity of "mushrooms," which they cooked and ate about six o'clock in the evening. The parents ate largely of the fungi, the two children merely dipping bread in the juice and eating the well-soaked bits. About midnight all members of the family were awakened by the most violent abdominal pain, accompanied by vomiting, headache, and extreme thirst. They were visited the following morning by a physician. He found them all seriously ill, the father profoundly prostrated, cyanotic, with muscular twitchings about the chest. The patient soon became delirious, with glazed eyes and contracted pupils. His symptoms were somewhat ameliorated during the day, but towards evening they returned with equal severity. For a period of eight days periodical attacks occurred finally resulting in coma and death. During the latter part of the illness the characteristic Hippocratic facies was present to an extreme degree. The mother suffered from corresponding symptoms, especially from the vomiting and thirst. She finally developed delirium and coma, and died on the eighth day. During her attack she aborted of a five months fœtus. Several similar cases are on record. The two children went into coma after a period of excessive pain and vomiting, dying 57 and 58 hours respectively after consuming the juice of the fungi. Specimens of the plants eaten were identified by botanists as *Amanita phalloides*.

### The Active Principle in the Fungus.

The active principle of *Amanita phalloides* has been sought from early times, but our first knowledge of it is due to Kobert, who, in 1891, described a hæmolytic poison in this fungus, considered it a "tox-albumin," and named it "*phallin*." This substance he believed to be the active principle. Kobert modified his views in 1900, when he described an additional poison of great strength, which he stated to be an alkaloid. Subsequently Ford confirmed these early observations as to the hæmolytic substances in *Amanita phalloides*, but on the basis of biological experiments was led to believe that an active toxine existed in the fungus, and to this body he gave the name *Amanita-toxin*. Recently Abel and Ford have pointed out that the hæmolytic principle is not a proteid (tox-albumin), but an easily decomposed glucoside. This glucoside, because of its extreme sensitiveness to heat, to small traces of acid, and to digestion by pepsin and pancreatin, can play no rôle in the intoxication in man where the cooked fungi are introduced into the stomach.

### Treatment.

No definite line of treatment can be recommended in *Amanita phalloides* intoxication beyond that adopted for poisoning in general. The thorough emptying of the stomach and intestines is usually brought about in the natural development of the intoxication, and should be encouraged rather than checked. Large quantities of morphia or some other anodyne are indicated to relieve the suffering and pain. The excessive thirst should be checked by the administration of any liquids which can be borne by the irritated stomach, and infusions of normal saline may be given subcutaneously. No drug has any antidotal effect upon the *Amanita-toxin*, and the only hope of successful treatment lies in the attempt to develop curative antitoxic sera, a procedure which Ford has shown to be theoretically possible.

### Poisoning by Amanita Muscaria.

The *Amanita muscaria*, known as the "fly-agaric" from the use of its decoction as a fly poison, is a beautiful mushroom, probably more generally recognised as poisonous than is the "deadly amanita." Poisoning from its ingestion is by no means uncommon, but the taste is so bitter that little of the fungus is as a rule swallowed, so that the mortality is low, many of the affected individuals recovering without untoward symptoms. The fungus when full grown is 6 to 8 inches high, with a widely expanded pileus. There is no distinct poison cup, the stem terminating in an expanded bulbous extremity. The stalk is covered with fairly adherent scales, which also lie thickly studded on the upper surface of the pileus. The stalk, the undersurface of the pileus, the gills, and spores are white. The top of the pileus is a beautiful yellowish or reddish-brown colour, varying in different countries.

### The Symptoms.

The symptoms of poisoning by *Amanita muscaria* are quite distinctive. They appear soon after the ingestion of the fungus, frequently within an hour or two. They consist of vomiting, diarrhœa, severe headache, with ocular symptoms, such as diplopia and contraction of the pupils, followed by delirium, a rapid loss of consciousness terminating in convulsions of the most violent character.

Death may result from the intoxication, but in the majority of cases only small bits of the fungus are swallowed, the rest being spat out on account of its bitterness, so that the effect of the poison gradually wears off. The delirium may be very marked and it is followed occasionally by amnesia.

In the cases which recover the serious symptoms ameliorate rapidly, and the period of convalescence, once established, is usually short—two or three days. Sometimes fungi either closely related to *Amanita muscaria*, or identical with it, lack the bitter principle of the true "muscaria," and are eaten in large quantity with rapidly fatal outcome.

### The Active Principle of Amanita Muscaria.

The active principle of *Amanita muscaria* is *muscarine*, a chemical substance related to neurine and choline, with the formula $C_5H_{15}NO_3$. It has been prepared synthetically by the oxidation of choline.

### Treatment.

As a logical consequence of our knowledge of the properties of muscarine, atropine was suggested as the physiological antidote, and it has been used with a considerable amount of success.

### Poisoning by Morells, or Helvellas.

According to Kobert there are no authentic cases of poisoning from the true Morells, of which he recognises five different species. There are, however, over 160 cases recorded of poisoning from the false Morells, properly called Lorcbells, of which the most important species is Helvella, or *Gyromytra esculenta*. Its active principle has been isolated and called helvellic acid. It is completely soluble in hot water, the aqueous extract killing dogs when given by the stomach. The symptoms of intoxication in these animals are the same as when the fresh fungus is eaten, and in both intoxications there is marked hæmoglobinuria, pointing to a hæmolytic poison. The toxicity of *Helvella esculenta* disappears, or is much lessened, when the fungi are dried.

Poisoning from Other Varieties of Fungi.

In addition to the species already described, the lethal effects of which are well established, a number of other fungi are credited with poisonous properties, and rarely death has been ascribed to their ingestion.

No cases of poisoning from the use of any of the purple-spored or black-spored agarics are on record. These plants, like many other foods when slightly decomposed, may give rise to severe intestinal disturbances, with marked prostration. The cases of poisoning reported from the field or meadow mushroom are not authentic. In the majority of instances the fungi said to be *Agaricus campestris* have not been identified as such by competent authorities, and where description of the plants has been given it is apparent that either *Amanita phalloides* or *Amanita muscaria* was eaten by mistake for the ordinary mushrooms.

Collectors of mushrooms for eating purposes should limit themselves to the use of well-known species, such as the purple-spored and black-spored agarics, the edible species of boletus, and certain varieties of lactarius and polyporus. White-spored agarics should always be avoided, even if the edible *Amanita cœsaria* or *aurantioca* is thus lost to the pleasure-loving palate. Especially should the *Lepiota* and the *Amanitopsis*, species closely allied to *Amanita phalloides*, be strictly eschewed; while the typical plants, perhaps, are not difficult to identify, specimens of *Amanita phalloides*, in which the veil has dropped off, closely resemble the *Amanitopsis*, and those in which the poison cup is sunk deep in the ground may readily be mistaken for *Lepiotas*. For an excellent list of edible and poisonous mushrooms those interested should consult the last edition of Kobert's "Lehrbuch der Intoxikationen."

